# Adhesive Properties of the Hyaluronan Pericellular Coat in Hyaluronan Synthases Overexpressing Mesenchymal Stem Cells

**DOI:** 10.3390/ijms21113827

**Published:** 2020-05-28

**Authors:** Sebastian Reiprich, Eva Hofbauer, Stefanie Kiderlen, Hauke Clausen-Schaumann, Wolfgang Böcker, Attila Aszódi, Veronika Schönitzer

**Affiliations:** 1 Experimental Surgery and Regenerative Medicine (ExperiMed), Department of General, Trauma and Reconstructive Surgery, Munich University Hospital, Ludwig-Maximilians-University, 80336 Munich, Germany; Sebastian.Reiprich@med.uni-muenchen.de (S.R.); eva.hofbauer@gmx.de (E.H.); Wolfgang.Boecker@med.uni-muenchen.de (W.B.); Attila.Aszodi@med.uni-muenchen.de (A.A.); 2 Center for Applied Tissue Engineering and Regenerative Medicine, Munich University of Applied Sciences, 80533 Munich, Germany; Stefanie.Kiderlen@googlemail.com (S.K.); hauke.clausen-schaumann@hm.edu (H.C.-S.); 3 Center for NanoScience, Ludwig-Maximilians-University, 80799 Munich, Germany

**Keywords:** hyaluronan, hyaluronan synthases, mesenchymal stem cells, early cell adhesion, stem cell niche

## Abstract

Hyaluronan (HA), a natural component of the extracellular matrix, is supposed to have a regulatory function in the stem cell niche. Bone marrow-derived human mesenchymal stem cells (hMSCs) are known to express all three hyaluronan synthases (HASes), which are responsible for HA production. HA is extruded into the extracellular matrix, but also stays bound to the plasma membrane forming a pericellular coat, which plays a key role during early cell adhesion. Since HAS isoenzymes, HAS1, HAS2 and HAS3, produce HA with different molecular weights, a difference in their role for cell adhesion is expected. Here, we transduced the immortalized hMSC cell line SCP1 to constitutively express eGFP-tagged HASes (SCP1-HAS-eGFP) by lentiviral gene transfer. The overexpression of the HAS-eGFP was shown on RNA and protein levels, HA was determined by ELISA and the stained HA-coat was analyzed using confocal microscopy. Time-lapse microscopy, spreading assay and single cell force spectroscopy using atomic force microscopy were applied to characterize adhesion of the different HAS transduced SCP1 cells. We showed in this study that HAS3 overexpressing cells formed the thickest pericellular coat compared with control or HAS1 and HAS2 transduced cells. Furthermore, SCP1-HAS3-eGFP displayed faster and stronger adhesion compared to cells overexpressing the other synthases or control cells. We conclude that overexpression of HASes in hMSCs differentially modulates their initial adhesive interactions with the substrate. This observation might be helpful in regenerative medicine goals.

## 1. Introduction

In comparison to other glycosaminoglycans, hyaluronan (HA) is a relatively simple molecule: it is non-sulfated, unbranched and not linked to any proteoglycan core protein. The molecule consists of alternating β-N-acetylglucosamine and β-d-glucuronic acid residues, and varies in its molecular weight ranging from 10^5^ to 10^7^ Da. Due to its carboxyl groups, hyaluronan is negatively charged at physiological pH and forms hydrated gels at low concentrations of <0.1%. Therefore, in the extracellular matrix, HA creates an ideal environment in which cells can migrate and proliferate [1,2]. HA also occurs in pericellular coats which were observed around different cell types like chondrocytes, fibroblasts [3] and mesenchymal stem cells [4]. HA coats are difficult to visualize since dehydration during sample preparation destroys the highly hydrated coat [5].

Hyaluronan synthases (E.C. 2.4.1.212) are the enzymes responsible for HA formation. They belong to the glycosyltransferases 2-family like chitin synthases and cellulose synthases. There are three known mammalian isoenzymes, HAS1, HAS2 and HAS3, which share a structural identity of 55–70%. They are all complex membrane proteins with 7 transmembrane domains and with an intracellular localization of the active site. In vitro, the HAS isoforms specifically synthesize HA of varying molecular weight: HAS1 and HAS2 polymerize HA chains of around 2 × 10^6^ Da, whereas HAS3 forms shorter chains of 1 × 10^5^ Da to 1 × 10^6^ Da [6,7,8]. The chain length influences HA function, therefore, the differential expression of the HAS can be critical for the cellular behavior [9,10].

Cell adhesion to the extracellular matrix (ECM) is critical for the regulation of cell proliferation, differentiation, motility and survival. In vitro adhesion studies indicate that cells undergo several steps until they firmly attach to and spread on the surface [11,12,13]. In various cell types one of the earliest steps of adhesion is mediated by the pericellular HA coat, which binds the cell to the surface but still keeping the cell membrane a few micrometers away from it [12,14]. Next, the transmembrane adhesion receptor integrins are activated and link the ECM components such as fibronectin and vitronectin to the actin cytoskeleton by intracellular binding partners like talin. Focal adhesion complexes containing paxillin, vinculin and talin are formed, which subsequently mature into focal adhesion sites and fibrillar adhesions, and the cell can spread on the substrate [11,15,16,17]. During the integrin-mediated adhesion stage, the cell membrane is only a few tens of nanometers away from the surface. Since the hyaluronan coat is usually thicker than this distance, the coat must be modified or removed at the transition phase of HA- and integrin-mediated adhesion [5].

Different cell types vary in the thickness of their pericellular coats: 4.4 ± 0.7 µm thick coat was determined around RCJ-P chondrocytes (rat chondrocytes from fetal calvaria) and 2.2 ± 0.4 µm thick coat was found around A6 epithelial cells using environmental scanning electron microscopy under fully hydrated conditions [18]. The thick coat of the chondrocytes is supposed to consist of an interweaved multilayer of HA chains, while the thinner coat of the epithelial cells is probably composed of a single layer in which each HA chain is directly anchored to the membrane [12]. The thick coat of the chondrocytes mediates initial “soft contacts” of the cells with the surface [18]. After 2–10 min, HA remains in discrete pockets between the cell and the surface and facilitates the formation of focal adhesion complexes [5]. In contrast, the thin hyaluronan coat of epithelial cells mediates rapid adhesion to the surface [14,19,20].

Human mesenchymal stem cells (hMSCs) are multipotent cells that can differentiate into muscle cells, chondrocytes, osteoblasts and adipocytes [21,22,23]. Due to their relatively easy isolation from many sources in the body and their in vitro expansion capacity, the therapeutic use of hMSCs is getting increasing credibility [24]. HMSCs are known to express all three HAS isoforms and CD44, a receptor for HA and a surface marker for hMSCs [4]. Furthermore, hMSCs are able to produce HA coats, which are supposed to be an important component of the stem cell niche due to its role in regulation of cell behavior [4,25,26].

Here, we generated immortalized hMSC cell lines each constitutively overexpressing one of the HAS isoenzymes with a C-terminal eGFP-tag in an active form. Since the three HAS isoforms produce HA with different molecular weights, we expect a difference in their role for cell adhesion. Modulation of the initial adhesive interactions of hMSCs in different ways may be helpful in recruiting hMSCs to injury sites. The capacity of hMSCs for regenerative medicine goals may be improved in this way.

## 2. Results

### 2.1. Generation of HAS-eGFP Overexpressing hMSCs

DNA fragments encoding human HAS1, 2 or 3 were fused with a C-terminal eGFP-tag by conventional PCR cloning methods. These constructs were cloned into the lentiviral expression plasmid pLenti4.3/V5-DEST for constitutive expression under the control of the CMV-promoter. Sequencing of the final expression plasmids revealed no mutation in the HAS and eGFP encoding regions. The plasmids were transferred into the well-established SCP1 hMSC cell line (hTERT immortalized bone marrow derived hMSCs [27]) by lentiviral gene transfer. As controls, SCP1 were transduced with the empty expression vector pLenti4.3-mock.

**Figure 1:**
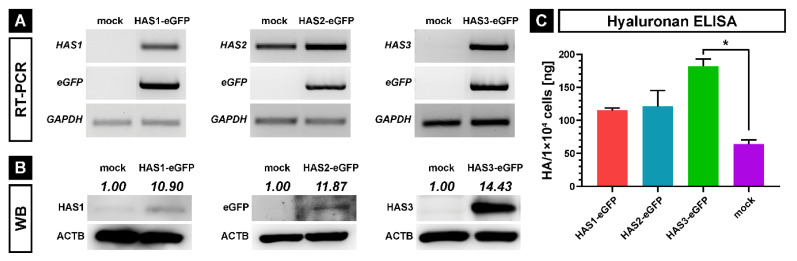
Analysis of HAS-eGFP overexpressing hMSCs (SCP1-HAS-eGFP) and control cells (SCP1-mock). (**A**) Expression analysis of *HAS-eGFP* mRNAs by semiquantitative RT-PCR in SCP1-HAS-eGFP and mock SCP1 cells. GAPDH (glyceraldehyde 3-phosphate dehydrogenase) was used as reference gene. (**B**) Western blot analysis of HAS-eGFP expressiosn in each of the cell lines; ACTB (actin beta) was used as loading control. Protein quantities were normalized to β-actin using Image J analysis software. (**C**) Hyaluronan ELISA assay-based quantification of secreted HA. The graph shows the mean HA content per 1 × 10^4^ cells in the supernatant after 48 h incubation in culture medium. Error bars represent SD, the asterisk indicates a *p*-Value < 0.05 (*).

The endogenous expression of HASes in SCP1 cells and the overexpression of each HAS isoform with an eGFP-tag (HAS-eGFP) were first confirmed on mRNA levels by RT-PCR (Figure 1A) using primer pairs specific for HASes and eGFP (Table 1). SCP1-mock cells moderately expressed HAS2, weakly HAS3, and did not express eGFP (Figure 1A). Of note, quantitative RT-PCR experiments were able to detect endogenous HAS1 mRNA in SCP1-mock cells with cycle threshold value of 35.In contrast, all SCP1-HAS-eGFP cell lines strongly expressed eGFP and the corresponding HAS cDNAs demonstrating successful overexpression of HAS1, HAS2 and HAS3 at the transcription level (Figure 1A). Next, western blotting was applied to verify protein expression using antibodies against HAS1, eGFP or HAS3 (Figure 1B). SCP1-mock cells showed weak signals for endogenous HAS1 and HAS3, while SCP1-HAS1-eGFP and SCP1-HAS3-eGFP cells strongly expressed HAS1 and HAS3 protein, respectively. As our HAS2 antibodies did not work on western blot, the expression of the HAS2-eGFP fusion protein in SCP1-HAS2-eGFP cells was confirmed by positive signal for the eGFP protein (Figure 1B, middle panel).

The ability of the transduced SCP1 cell lines to oversecrete HA into the supernatant was determined by HA-specific ELISA after 48 h cultivation in the presence of 2.5 mM UDP-N-acetyglucosamine and 2.5 mM UDP-D-glucuronic acid. In comparison to the control SCP1 mock cells, SCP1-HAS1-eGFP and SCP1-HAS2-eGFP cells showed moderately increased (1.8-fold), while SCP1-HAS3-eGFP cells strongly elevated (2.8-fold) secretion of unbound HA in the medium (Figure 1C).

In summary, all three SCP1-HAS-eGFP cell lines oversecrete HA, therefore, they express the transgene proteins in their active form. Since it is known that only HASes localized in the plasma membrane are active [28], these results also indicate that the HAS-eGFP fusion proteins are correctly transported to the plasma membrane. In our experiments, SCP1-HAS3-eGFP cells exhibited the highest expression level and produced and secreted the most HA.

### 2.2. The Pericellular Coat on HAS-eGFP Overexpressing hMSCs

**Figure 2:**
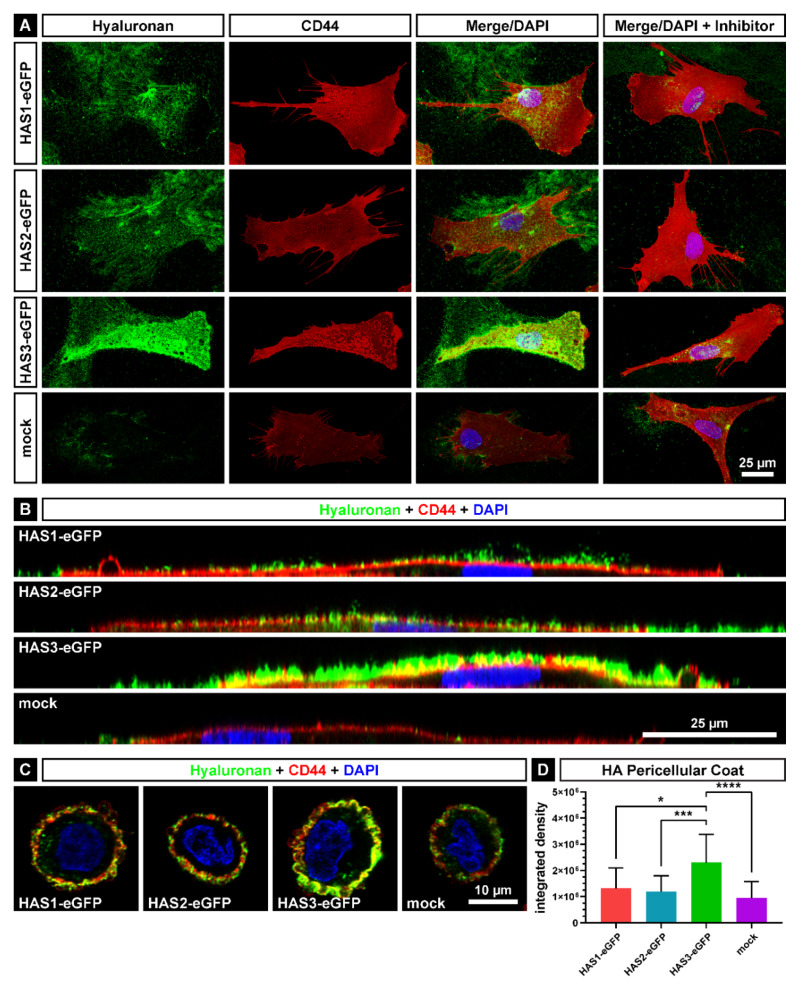
Analysis of the HA pericellular coat. Confocal microscopy of SCP1-HAS-eGFP and SCP1-mock cells with immunofluorescently labelled HA (green colored) and CD44 (red colored). Nuclei were counterstained with DAPI (blue). (**A**) z-stack maximum projection of cells incubated 48 h in culture medium containing 10 mM GlcNAc and cells additionally incubated with 1 mM of the HAS-inhibitor 4-MU as control. (**B**) Optical sections of the y-stack of the non-inhibited cells from Figure 2A. (**C**) HA pericellular coat on detached cells. Confocal microscopy of SCP1-HAS-eGFP and SCP1-mock cells detached by accutase. Optical sections of the z-stack of cells incubated for 24 h in culture medium with 10 mM GlcNAc and in the following 24 h under serum-free conditions. (**D**) Integrated density as quantification of the HA fluorescence signal in detached SCP1-HAS-eGFP and SCP1-mock cells. Error bars represent SD, the asterisks indicate a *p*-Value < 0.05 (*), < 0.001 (***) or < 0.0001 (****).

For the visualization of the membrane-bound, pericellular HA, monolayer cultured HAS-eGFP overexpressing SCP1 and control cells were fixed and incubated with biotinylated HA-binding complex, followed by staining with Alexa Fluor 647-conjugated streptavidin. In parallel, fluorescence immunostaining was used to visualize the HA receptor CD44. Imaging was performed using confocal laser scanning microscopy (Figure 2). A green colored HA coat on the plasma membrane was detected in all HAS-eGFP transduced cell lines (Figure 2A). The highest fluorescence signal intensity was seen in SCP1-HAS3-eGFP cells, while SCP1-HAS1-eGFP and SCP1-HAS2-eGFP cells exhibited less intense HA coat. In contrast to SCP1-HAS-eGFP cells, SCP1-mock cells showed a very weak fluorescence signal intensity. Interestingly, the three SCP1-HAS-eGFP cell lines revealed HA “footprints” on the surface, which probably are membrane/HA-coat leftover patches of the migrating cells (Figure 2A). When cells were cultured in presence of HAS inhibitor 4-methylumbelliferone before staining, little, if any Alexa Fluor 647 fluorescence signal was detected confirming the specificity of the HA staining (Figure 2A). All cell lines, including SCP1-mock, expressed a high level of CD44 on the plasma membrane. The side view indicated that the CD44 staining is located in the plasma membrane (Figure 2B). The vertical profile shows the pericellular location of the HA in the HAS-eGFP overexpressing hMSCs (Figure 2B). In comparison to SCP1-mock, the SCP1-HAS-eGFP cells formed a thicker hyaluronan pericellular coat. In accordance to the highest mRNA and protein expression levels in HAS3-eGFP transduced cells (Figure 1A,B), HA seemed to cover the plasma membrane most densely in SCP1-HAS3-eGFP cells (Figure 2B).

The difference in the thickness of the pericellular HA coat was also visualized and quantified by HA staining of suspended cells, which were detached from the surface of the plastic culture dish by accutase treatment (Figure 2C,D). Imaging of single confocal stacks demonstrated the presence of a thicker HA coat around SCP1-HAS-eGFP cells compared with SCP1-mock cells (Figure 2C). Quantification of the HA coat was performed by measuring the integrated density of the HA’s fluorescence signal around the SCP1-HAS-eGFP and SCP1-mock cells. Significantly stronger signal was detectable for SCP-1-HAS3-eGFP cells compared to the three other cell lines (Figure 2D). Compared to SCP1-mock, HAS transduction induced 1.39-, 1.25- and 2.42-fold increase in HA coat thickness for HAS1, HAS2 and HAS3, respectively.

### 2.3. The Adhesion Properties of HAS-eGFP Overexpressing hMSCs to Tissue Culture Polystyrene Surface

**Figure 3:**
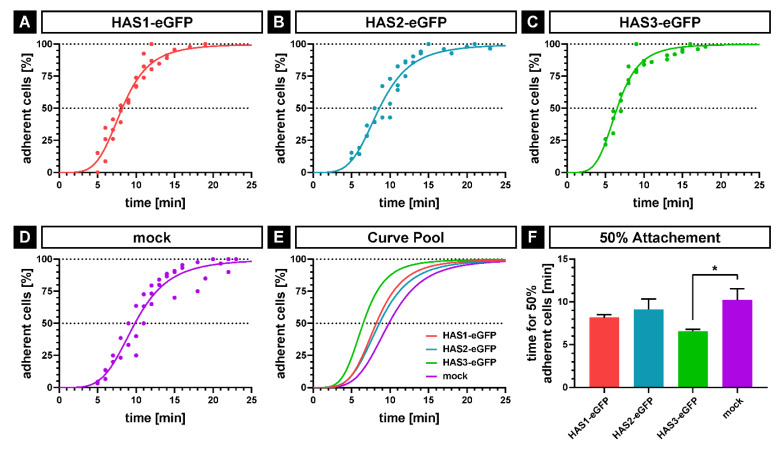
Analysis of initial cell attachment by time-lapse microscopy. SCP1-HAS-eGFP and SCP1-mock cells were incubated for 48 h in culture medium in the presence of 10 mM GlcNAc including the last 24 h under serum-free conditions. The cells were detached by accutase treatment and were seeded onto uncoated tissue culture polystyrene dishes. Cell attachment was determined by the formation of the first protrusion and the whole process was imaged with 60 frames/h. (**A–D**) Nonlinear regression by sigmoidal four-parameter-logistic; dots indicate the adhesion of a minimum of one cell at the corresponding time point, showing values of three independent experiments. (**E**) Overlay of the nonlinear regression curves of the four cell lines. (**F**) Mean values for 50% adherent cells calculated by sigmoidal four-parameter-logistic for each of the three independent experiments. Error bars represent SD, the asterisk indicates a *p*-Value < 0.05 (*).

As evidence from previous studies has indicated that the HA coat mediates early steps of cell-surface interactions [21,22,23], we have analyzed the influence of HAS-eGFP overexpression on adhesive properties of the transduced SCP1 cells. The initial adhesion of HAS-eGFP overexpressing hMSCs and SCP1-mock cells to tissue culture polystyrene (TCPS) surface was analyzed by time-lapse microscopy under serum-free conditions. Adherent cells were identified by the appearance of the first cell protrusion, and the percentage of adherent cells were determined at each time point. The adhesion kinetics of all cell lines showed a similar trend: an initial non-adherent phase (1–4 min); an exponential attachment phase (about 10–20 min) and a plateau phase when all seeded cells are adhered (Figure 3A–E). In accordance to the thickness of their pericellular HA coat, the curve progression of SCP1-HAS1-eGFP and SCP1-HAS2-eGFP was similar. The SCP1-HAS3-eGFP cells, which showed the most bounded HA, adhered faster than the other cell lines. The SCP1-mock cells, which have very little pericellular HA coat, adhered slowest. In the case of SCP1-mock, 50% of the cell population adhered to the uncoated surface in 10.2 min ± 1.3 (SD; *n* = 3 experiments). 50% adhesion was achieved after 8.2 min ± 0.4 in the case of SCP1-HAS1-eGFP cells, after 9.1 min ± 1.2 in the case of SCP1-HAS2-eGFP cells and 6.6 min ± 0.2 in the case of SCP1-HAS3-eGFP. The adhesion kinetic of SCP1-HAS3-eGFP was significantly accelerated (1.54-fold faster) compared to SCP1-mock (Figure 3F, *p* < 0.05). The experiment was also done on surfaces coated with the bone matrix proteins type I collagen and fibronectin, however, both HAS-transduced and SCP1-mock cells adhered to the substrate very fast after plating, which prevented recording the adhesion kinetics with our assay.

**Figure 4:**
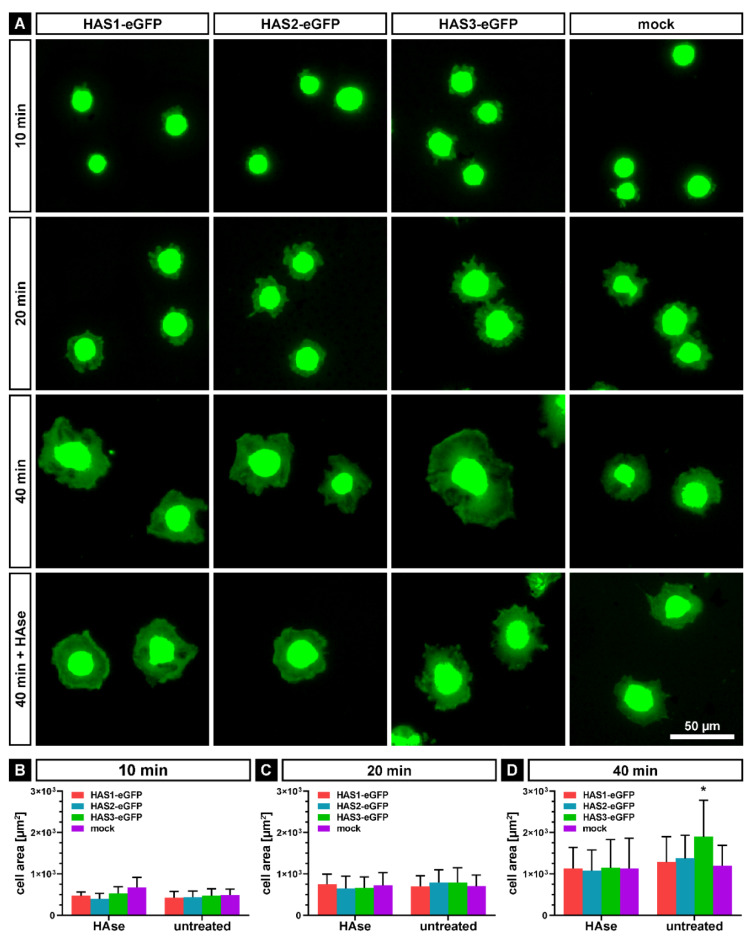
Spreading of SCP1-HAS-eGFP and SCP1-mock cells on tissue culture polystyrene surface. Cells without or with hyaluronidase (HAse) treatment were seeded under serum-free condition onto the surface of tissue culture dishes and cultured for 10, 20 and 40 min. (**A**) Representative fluorescence micrographs of cells stained with BODYPY 581/591 SE. Mean cell area at 10 (**B**), 20 (**C**) and 40 min (**D**) after seeding compared to HAse treated control cells. Error bars represent SD, the asterisk indicates a *p*-Value < 0.05 (*) in comparison to all other data sets in D.

Following the initial adhesion to the TCPS surface, the cells proceed to the “active event” of spreading. In order to further analyze adhesion and spreading, SCP1-HAS-eGFP and SCP1-mock cells were plated on tissue culture dishes in serum-free medium; fixed in 4% PFA after different time points (10, 20 and 40 min) and stained with BODIPY 581/591 SE to visualize the cells. After imaging, the cell area was quantified using FIJI software. As a control, cells were treated with hyaluronidase (HAse) for 1 h before plating. After 10 min of incubation, the most adhered cells were small and roundish, and now obvious difference in the cell areas was observed between HAS-eGFP transduced and SCP1-mock cells (Figure 4A,B). At 20 min, the cells showed spreading, and the mean cell areas were comparable between HAS overexpressing and mock cells (Figure 4A,C). HAse treatment did not influence cell spreading at 10 and 20 min (Figure 4B,C). After 40 min, the cell areas of SCP1-HAS1-eGFP and SCP1-HAS2-eGFP cells were mildly increased in comparison to SCP1-mock cells. SCP1-HAS3-eGFP cells demonstrated the largest, 1.4-fold increase in the mean cell area compared to SCP1 control cells (Figure 4A,D). HAse digestion decreased the spreading area to the same levels in each experimental group compared with the untreated groups (Figure 4A,D).

Taken together, our observations imply that an increase in the HA coat, especially by overexpression of HAS3-eGFP, accelerates the initial adhesion and subsequent spreading of SCP1 cells on the surface of plastic tissue culture dish.

### 2.4. Focal Adhesion Formation in HAS-eGFP Overexpressing hMSCs during Attachment and Spreading

**Figure 5:**
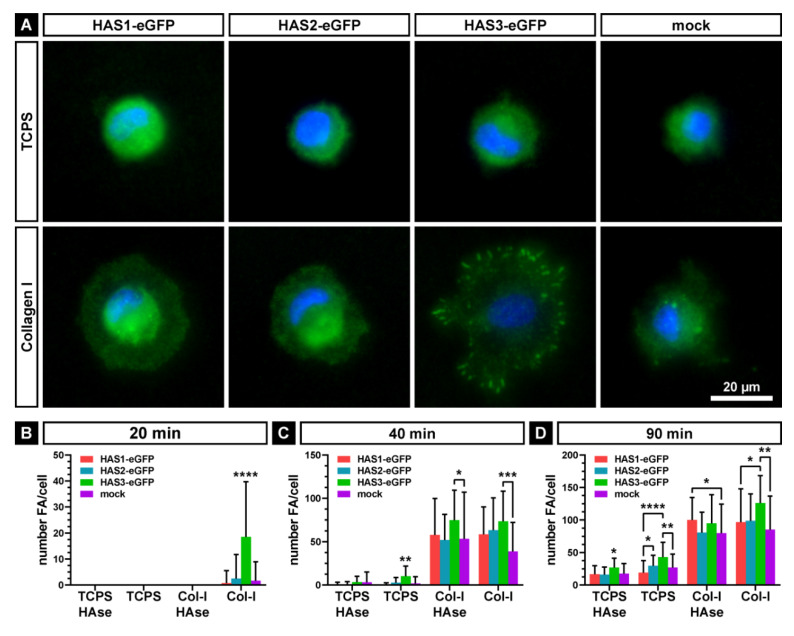
Formation of vinculin-containing focal adhesions (FA) of SCP1-HAS-eGFP and SCP1-mock cells. Cells without or with hyaluronidase (HAse) treatment were seeded under serum-free condition onto the surface of tissue culture dishes (TCPS) or type I collagen (Col-I) and cultured for 20, 40 and 90 min. (**A**) Representative fluorescence micrographs of untreated cells stained for vinculin after 20 min of adhesion. Nuclei are counterstained with DAPI. Mean number of focal adhesions at 20 (**B**), 40 (**C**) and 90 min (**D**) after seeding. Error bars represent SD, the asterisks indicates a *p*-Value < 0.05 (*), < 0.01 (**), < 0.001 (***) or < 0.0001 (****). Without brackets they indicate a significant difference of the labeled dataset compared to all others of the same group.

Next, we investigated the effect of the pericellular HA coat of SCP1-HAS-eGFP and SCP1-mock cells on focal adhesion formation during attachment and spreading. As control, cells were treated with hyaluronidase for reduction of the HA-coat. Of note, we were still able to detect some residual fluorescence signal in the staining of the coat of SCP1-HAS3-eGFP cells. The untreated and hyaluronidase-treated cells were then seeded under serum-free conditions on TCPS surface and on Col-I-coated surface. After 20, 40 and 90 min, the cells were fixed and the formation of vinculin-containing, integrin-mediated adhesion structures was analyzed by immunostaining. After 20 min, hyaluronidase-treated cells lacked distinct vinculin-stained structures on TCPS indicating that the presence of an ECM on the surface is pivotal for the formation of focal adhesions. In contrast, on Col-I-coated surface the cells started to develop focal adhesions, which were, hovewer, not observed after HAse treatment suggesting a role of the HA coat for the initiation of attachment complexes. In comparison to the other cell lines, SCP1-HAS3-eGFP with the thickest HA-coat formed significantly more vinculin-containing structures on Col-I-coated surface (Figure 5A,B). Under this condition, 52% of SCP1-HAS3-eGFP cells formed vinculin-containing, large focal adhesions at the cell periphery. Only 3% of SCP1-HAS1-eGFP, 8% of SCP1-HAS2-eGFP and 5% of SCP1-mock showed these structures. Upon longer incubation time (40 and 90 min after seeding), cells formed focal adhesion structures under all conditions (Figure 5B), but they were more prominent on Col-I-coated surface than on TCPS surface. The appearance of vinculin-rich structures on TSCP suggests that the cells produced their own matrix promoting the development of focal adhesions. The presence of focal, vinculin positive areas in all HAse-treated cell types indicates that the HA-coat apparently does not play a key role in the maturation of the focal adhesion structures during spreading. However, SCP1-HAS3-eGFP cells showed an increased number of focal adhesions compared to mock and HAS1 or HAS2 overexpressing cells.

Thus, we conclude that the thicker HA coat of the SCP1-HAS3-eGFP cells can mediate the formation of focal adhesion structures during the early cell-matrix adhesion at the very first contact with the substrate. During spreading, the HA-coat has no significant effect on the maturation of the focal adhesions.

### 2.5. Single Cell Force Spectroscopy of HAS-eGFP Overexpressing hMSCs

**Figure 6:**
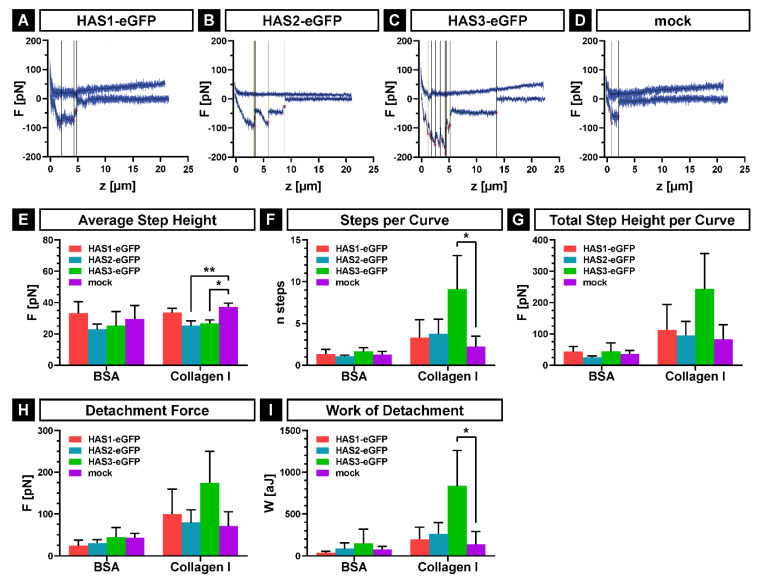
Adhesion forces of SCP1-HAS-eGFP and SCP1-mock to Col-I and BSA coated surfaces. Cells were incubated 24 h in culture medium containing 10 mM GlcNAc prior to AFM measurements. The experiments were performed in serum-free HEPES-buffered MEM α medium with 10 mM GlcNAc at 37 °C. (**A–D**) Representative force-distance curves for each of the cell lines; vertical lines indicate single step positions. (**E**) Average step heights show the mean force necessary to detach one adhesive event. (**F**) Steps per curve indicate the mean number of adhesive events per cell. (**G**) The overall adhesive force per cell is shown by the total step height per curve. (**H**) The detachment force is defined as the global maximum of a detachment force curve. (**I**) Work of detachment was calculated as the integral between the detachment force-distance curve and its corresponding base line. Error bars represent SD, the asterisks indicate a *p*-Value < 0.05 (*) or < 0.01 (**).

In order to quantify the adhesion forces between the single cell of SCP1-HAS-eGFP or SCP1-mock and the bone matrix protein collagen-I (Col-I) as well as the negative control bovine serum albumin (BSA), we used atomic force microscopy (AFM) based single cell force spectroscopy as described elsewhere in detail [29,30,31]. First, a single cell was picked up from the non-adhesive BSA-coating with a tip-less, poly-L-lysine-coated AFM cantilever. Afterwards, the cell was brought into contact with Col-I- or BSA-coated cell culture dishes until a predefined contact force (100 pN) was reached. Subsequently, the force necessary to detach the cell from the substrate was recorded resulting in force-distance plots. Figure 6A–D show typical force distance curves of each cell lines on the Col-I-coated substrate, indicating stronger de-adhesion events for SCP1-HAS3-eGFP compared to the other cell lines. The evaluation of these curves confirms the highest affinity of SCP1-HAS3-eGFP cells to Col-I. The average step height between the cell lines showed no differences related to the used substrate, but stronger adhesive forces for single steps in the mock cells compared to SCP1-HAS2-eGFP and SCP1-HAS3-eGFP on Col-I (Figure 6E). Using Col-I as substrate, SCP1-HAS3-eGFP showed an increased number of steps per curve (Figure 6F) and an increased total step height per curve (Figure 6G) in comparison to the other cell lines. In order to detach a single SCP1-HAS3-eGFP more steps with higher force application are necessary than to detach single cells of SCP1-HAS1-eGFP, SCP1-HAS2-eGFP or SCP1-mock. In addition, the maximum detachment force (global maximum of detachment force curve, Figure 6H) and the adhesion energy (work of detachment, integral between baseline and force curve, Figure 6I) showed the same tendency with the highest values for SCP1-HAS3-eGFP compared to the other two isoforms and SCP1-mock. The control measurements on surfaces treated with BSA revealed the weakest interactions for all cell lines (Figure 6F–I).

Altogether, the results reveal the SCP1-HAS3-eGFP cell adhesion to Col-I as the strongest of the analyzed adhesion forces.

## 3. Discussion

HMSCs are still of major interest in the use of regenerative medicine because of their non-invasive accessibility and their differentiation capacity into various cell types. The immortalized hMSCs (SCP1) used in this study have been previously shown to have the ability to differentiate into the chondrogenic, adipogenic and osteogenic lineages in vitro [27] and have been successfully used in a bone tissue engineering study [32].

However, in order to fully exert the potential of hMSCs, their efficient recruitment and adhesion toward lesion sites are essential [33]. The results of the present study demonstrate that overexpression of HASes, mainly HAS3, is able to increase the HA coat and improve the adhesive properties of hMSCs.

HMSCs are known to produce HA themselves. Qu et al. [4] detected the expression of all HAS isoforms in hMSCs of different donors, and found that HAS2 being the most abundant isoform. Consistently, in our study we could mainly detect expression of HAS2 in the control SCP1-mock cells, alongside with very low expression levels for HAS1 and HAS3. Furthermore, in comparison to the HAS-eGFP overexpressing cells, the SCP1-mock produced less HA demonstrated by ELISA and staining of the cell-bound HA. The overexpression of hTERT of the immortalized SCP1 hMSCs might have an impact on the expression pattern of the HASes, since their expression levels are changed during senescence [34]. A further explanation might be that we did not add FGF-2 into the culture media, which is known to stimulate the expression of HASes and the production of HA [35]. Another previous study demonstrated a higher HAS1 mRNA expression level in bone marrow derived hMSCs suffering from multiple myeloma than in hMSCs from healthy donors. In accordance with our data, HAS2 was the dominated isoform in the normal hMSCs [36].

Here, we observed an increased HA content in the supernatant of SCP1-HAS-eGFP compared to the SCP1-mock indicating the correct insertion of all HAS-eGFP transgene and the directional transport of HA through the plasma membrane. However, as the smallest transmembrane protein, HAS3-eGFP (58 kDA; in comparison: HAS1-eGFP: 92 kDa; HAS2-eGFP: 90 kDa) seems to have the highest expression efficiency as well as the highest activity. The HAS-eGFP overexpressing cells produce HA in a range between 115 and 182 ng/10,000 cells. SCP1-mock secreted 1.8–2.8 fold less HA in the culture medium (64 ng/10,000 cells). Qu et al. [4] detected similar values of secreted HA ranging from 22.5 to 397.4 ng/10,000 cells of hMSCs from different donors. However, the production of HA was strongly donor dependent. On accordance to our study, HAS3 overexpressing Chinese Hamster Ovary cells also produced more HA than HAS1 and HAS2 overexpressing cells [37]. 

In addition, the staining of the HA-coat confirmed that the SCP1-HAS3-eGFP had the thickest coat followed by the SCP1-HAS2-eGFP and the SCP1-HAS1-eGFP. The cell lines might differ in the thickness of the pericellular coats shown by immunofluorescence due to different amounts of formed HA or due to differences in HA to receptor interactions. Our immunofluorescence analysis confirmed that all cell lines established in this study express CD44 in great amounts. CD44 is known to be the major receptor of HA and to be responsible for keeping HA in the pericellular matrix [4,38]. Interestingly, SCP1-HAS3-eGFP, which seems to have the thickest coat, showed short plasma membrane protrusions also indicated by the spotted appearance of the CD44 staining. However, we do not see the extensions as distinct as they appeared in previous studies [4,38].

Evidence from previous studies indicated that the pericellular HA coat of chondrocytes and epithelial cells effect initial cell attachment [5,12,13,14,18]. Here, we demonstrated that the early adhesive properties of SCP1 also differ depending on the amount of plasma membrane-bound HA. A positive correlation between HA amount and the adhesion rate could be observed. SCP1-HAS3-eGFP showed the highest amount of HA as well as the greatest attachment rate, whereas SCP1-mock revealed the lowest amount of HA and the slowest attachment rate on tissue culture dishes. Our time-lapse microscopy experiments revealed that 50% of the cells were attached and started flattening within 6.5 min (SCP1-HAS3-eGFP) to 10.2 min (SCP1-mock). Complete cell spreading took over 40 min, which was enhanced by the thick HA coat for the SCP1-HAS3-eGFP cells, and partially inhibited by the removal of the HA coat with HAse treatment. Similar time scales of adhesion and spreading were described for epithelial A6 cells in the literature [14]. The analysis of the adhesion by time-lapse microscopy and the spreading assays were performed with untreated cell culture dishes. When we coated the dishes with Col-I or fibronectin, the cells were immediately adherent after plating. Therefore, we were not able to perform the classical adhesion assay with other substrates on the surface, which might be a better model for the natural surface of bone.

The immunostaining of vinculin-containing structures showed that SCP1-HAS3-eGFP cells display the highest number of focal adhesions during early attachment on Col-I coated surface. Removal of the hyaluronan-coat seems to delay the formation of focal adhesion structures suggesting that HA provoke the first contact with the substrate and facilitates the formation of vinculin-containing, integrin-mediated adhesions. Consistently with our results, Cohen et al. [5] demonstrated that the pericellular HA-coat of chondrocytes modulates the formation of focal adhesions. As possible mechanisms for the transition of the hyaluronan-mediated to the integrin-mediated adhesion, the authors suggest that the interaction of the hyaluroan-coat with the surface might trigger adhesion signaling events e.g., via the hyaluronan receptor CD44. Our single cell force spectroscopy data, however, demonstrate that SCP1-HAS3-eGFP cells exhibit the highest early adhesion characteristics against Col-I before focal adhesion becomes engaged. These results indicate that the HA coat influences hMSC attachment to the main organic component of the bone surfaces.

Altogether, the results from the present study demonstrate that the modulation of the HA-coat by overexpression of HASes accelerates the attachment of human mesenchymal stem cells to tissue culture polystyrene surfaces, and strengthens their adhesion to collagen-I-coated surface. In vivo, the HA-coat is supposed to be an important component in the stem cell niche by regulating cell behavior through binding to cell surface receptors [2,39] or signal-transducing receptors [25,40]. Our data suggest, that the HA-coat might be responsible for the homing and residence of the stem cells in their niche. It might also play an important role in stem cell differentiation regulation, since the HA-coat correlates with the differentiation status of stem cells and progenitor cells [4,40,41]. Further investigations would be necessary to address the impact of the pericellular HA coat itself on the differentiation potential of SCP1-HAS-eGFP cells. However, modulating the size of the HA-coat in hMSCs might be helpful in regenerative medicine goals.

## 4. Materials and Methods

### 4.1. Cloning of HAS-eGFP and Transduction of hMSCs

The cDNA sequence of each of the three HASes (Source BioScience LifeSciences, Nottingham, UK) was ligated in the PCR2.1-TOPO vector (Invitrogen, Darmstadt, Germany) to transfer it via restriction and ligation into a pENTR11 backbone (Invitrogen), fusing the synthases to the 5′-end of an eGFP sequence already present in the vector. Finally, the HAS-eGFP sequence was transferred by homologous recombination into a pLenti4.3/V5-DEST plasmid (Invitrogen) containing the CMV promotor and a Zeocin selection marker for stable selection. In parallel, a mock pLenti4.3 plasmid without expression cassette was created. The final plasmids were controlled by DNA sequencing (Eurofins MWG Operon, Ebersberg, Germany). For the lentivirus generation, the pLenti4.3 constructs were isolated using the Endo-Free Plasmid Mini Kit II (Qiagen, Hilden, Germany). Afterwards, the lentiviral particles were produced in 239FT cells (Invitrogen) using the ViraPower Lentiviral Expression System (Invitrogen). The hTERT-expressing hMSC line SCP1 [27] was transduced with the obtained viral particles to receive three HAS-overexpressing cell lines and a control cell line only transduced with the empty vector.

### 4.2. Cell Culture Conditions

SCP1 cells were cultivated in a culture medium consisting of MEM α with nucleosides and GlutaMAX supplement (Life Technologies, Darmstadt, Germany) containing 10% (*v/v*) fetal bovine serum (Sigma-Aldrich, St. Louis, MO, USA) and 1000 U/mL penicillin and 1000 µg/mL streptomycin (Biochrom, Berlin, Germany) at 37 °C with 5% CO_2_ and ~90% humidity. After transduction, additional 100 µg/mL Zeocin (Invitrogen) was added to the medium for selection and to maintain the HAS-eGFP-overexpression.

### 4.3. Reverse Transcriptase-Polymerase Chain Reaction (RT-PCR)

RNA isolation from cells was performed using the RNeasy Mini Kit (Qiagen) as recommended by the manufacturer when cells formed confluent monolayers on T-25 culture flasks (Nunc, Darmstadt, Germany). To obtain cDNA, a Reverse Transcriptase-polymerase chain reaction (RT-PCR) was carried out using the Transcriptor First Strand cDNA Synthesis Kit (Roche, Penzberg, Germany) according to the manufacturer’s instruction. The expression levels of the three isoforms were controlled by semiquantitative PCR using 2.5 units FastStart Taq DNA Polymerase (Roche) with the supplied buffer and nucleotides, sense and antisense primers (10 pM each, Table 1, Life Technologies, Darmstadt, Germany) and 100 ng cDNA in a reaction volume of 25 µL. Afterwards, the PCR products were analyzed by agarose gel electrophoresis. The housekeeping gene GAPDH was used as control.

### 4.4. Western Blot

For protein isolation, cells were cultured in T-75 flasks (Nunc) under standard conditions until confluence. After detachment by trypsin-EDTA (PAA, Pasching, Austria), cells were pelleted by centrifugation for 5 min at 500 rcf, washed in 5 mL Dulbecco’s phosphate buffered saline (DPBS, Biochrom) and resuspended at a concentration of 2 × 10^7^ cells/mL in RIPA buffer (0.1% sodium dodecyl sulfate (SDS), 1% NaDOC, 1% Triton X-100, 50 mM Tris-HCl pH 8.2, 150 mM NaCl, 10 mM EDTA, 20 mM NaF) containing protease inhibitor (Roche). After an incubation for 30 min on ice, the cells were lysed by the freeze-thaw method. The lysates were centrifuged at 4 °C with 10^4^ rcf for 10 min. The supernatant was used for further investigation. The protein concentration was determined by the Micro BCA Protein Assay Kit (Thermo Fisher, Schwerte, Germany) according to the manufacturer’s instructions. 40 µg of protein were mixed with one third of their volume of 4× Laemmli buffer (200 mM Tris-HCl pH 6.8, 40% glycerol, 10% SDS, 30% β-mercaptoethanol, 0.02% bromphenolblue) and denatured for 5 min at 95 °C. These lysates were separated by SDS-PAGE on 8% gels and transferred onto a PVDF membrane. Blocking was performed in 5% skim milk powder (Merck, Darmstadt, Germany) in Tris buffered saline with Tween (TBST, 10 mM Tris-HCl pH 7.4, 150 mM NaCl, 0.05% Tween20) for 1 h. The following primary antibodies were applied: rabbit anti-HAS1 (ab128321, Abcam, Cambridge, UK), rabbit anti-HAS3 (266–281, Sigma-Aldrich), rabbit anti-GFP Epitope Tag (Dianova, Hamburg, Germany) and mouse anti-β-actin (Santa Cruz Biotechnology, Santa Cruz, CA, USA). Primary antibodies were diluted in 5% milk in TBST and were incubated with the membrane at 4 °C overnight. Afterwards, either goat anti-rabbit-HRP (Santa Cruz Biotechnology, Santa Cruz, CA, USA) or goat-anti-mouse-HRP (Rockland, Gilbertsville, PA, USA) was used as secondary antibody. Also the secondary antibodies were diluted in the same manner and incubated for 1 to 1.5 h at room temperature. Protein visualization was carried out in an ImageQuant LAS 4000 mini (GE Healthcare, Munich, Germany) using Luminata Classico/Crescendo Western HRP Substrate (Millipore, Billerica, MA, USA).

### 4.5. HA-ELISA

To analyze the HAS activity in the SCP-HAS-eGFP, 9500 cells were seeded per well of a 24-well plate (Nunc, Darmstadt, Germany) in duplicates. As substrates, 2.5 mM of uridine 5′-diphospho-N-acetylglucosamine sodium salt (Sigma-Aldrich) and 2.5 mM uridine 5′-diphosphoglucuronic acid trisodium salt (Sigma-Aldrich) were added to the standard culture medium. After 48 h incubation, the supernatant was collected and the cells were counted. The quantification of the HA content in the supernatant was performed by the commercial enzyme-linked immunosorbent assay (ELISA) TECO Human Hyaluronic Acid Test TE1017 Kit (TECOmedical, Bünde, Germany) according to the manufacturer’s instructions and quantified in a photometric plate reader MultiscanFC (Thermo Scientific). Four wells per experiment were measured, the whole experiment was performed twice.

### 4.6. HA Coat Immunostaining

To visualize the HA coat, a fluorescence staining was performed. Therefore, 500 cells were seeded in 300 µL culture medium containing 10 mM N-acetyl-D-glucosamine (GlcNAc, Sigma-Aldrich,) per well of an 8-well chamber slide ibidiTreat (Ibidi, Planegg, Germany). As a control, 1 mM 4-methylumbelliferone (Tokyo Chemical Industry, Eschborn, Germany) was added to inhibit the HAS’activity. After 48 h cultivation, the cells were washed with 300 µL/well DPBS and fixed for 10 min at room temperature with 300 µL 4% paraformaldehyde in DPBS. After an additional washing step with DPBS, the cells were blocked with 300 µL 1% (*w/v*) BSA (Sigma-Aldrich) in DPBS for 1 h at room temperature. Following, 5 µg/mL of a biotinylated hyaluronic acid binding protein (HABP, Merck Millipore, Darmstadt, Germany) and a mouse anti-CD44 antibody (1:400, #3570, Cell Signaling Technology, Cambridge, UK) were diluted in 1% BSA in DPBS. The fixed cells were incubated over night at 4 °C in 100 µL of the primary solution per well. Afterwards, the wells were washed with 300 µL of 1% BSA in DPBS. As secondary antibodies, a donkey anti-mouse Alexa Fluor 546 conjugate (4 µg/mL, A10036, Thermo Fisher Scientific) and a streptavidin Alexa Fluor 647 conjugate (10 µg/mL, S21374, Thermo Fisher Scientific) diluted in 1% BSA in DPBS were added for 1 h at room temperature. The cells were washed again with 300 µL DPBS before the nuclei were counter-stained for 2 min with 4,6-diamidino-2-phenylindole (DAPI) and washed a last time with DPBS. Finally, the stained cells were kept in DPBS to perform confocal microscopy using a Leica SP8 AOBS WLL, a HC PL APO 63×/1.30 GLYC CORR CS2 objective and Lightning deconvolution software applying 1.28× zoom (all from Leica Microsystems, Wetzlar, Germany).

### 4.7. Time-Lapse Adhesion Assay

The SCP1-HAS-eGFP and the SCP1-mock were incubated for 24 h in culture medium containing 10 mM GlcNAc and afterwards 24 h in serum-free culture medium containing 10 mM GlcNAc. For detachment of the cells, StemPro Accutase (Thermo Fisher Scientific) was used. The reaction was stopped with serum-free culture medium containing 10 mM GlcNAc and washed in the same medium. To perform the adhesion assay, 1.8 × 10^4^ cells were seeded on Nunclon Delta Surface 6-well plates (Nunc) in 4 mL. The adhesion was imaged with 60 frames/h for 150 min using an inverted microscope Axiovert S100 (Carl Zeiss, Oberkochen, Germany) with a 20× phase contrast objective equipped with a controllable biochamber (Pecon, Erbach, Germany). For every cell, the time point was determined when they formed the first visible protrusion differing from the round shape of unattached cells using FIJI software [42]. By applying a sigmoidal four-parameter-logistic in GraphPad Prism software (GraphPad Software, San Diego, CA, USA), the time point of 50% adherent cells was calculated. The experiment was performed three times with a mean value of 32 cells per line and repetition.

### 4.8. Spreading Assay

Cells were preincubated in culture medium containing 10 mM GlcNAc as described above. Control cells were treated with 500 U/mL hyaluronidase (HAse from bovine testes, Sigma-Aldrich) for 1 h at 37 °C and an additional washing step. Afterwards, cells were seeded on Nunclon Delta Surface 6-well plates (Nunc) in the following densities: 2000, 4000 and 10,000 cells/cm^2^. 10, 20 and 40 min after seeding, the cells were washed two times with DPBS and fixed with 4% paraformaldehyde in DPBS. The 6-well plates were stained with BODIPY 581/591 SE (Invitrogen, Darmstadt, Germany), a dye binding to free amino groups via NHS esters. Per cell line, treatment and time point between 38 and 97 cells were imaged with an AxioObserver Z1 (Carl Zeiss) and their area was measured using the FIJI software [42].

### 4.9. Quantification of Focal Adhesions

To quantify the formation of focal adhesions, the cells were preincubated in culture medium containing 10 mM GlcNAc and HAse-treated control cells were created as described above. The cells were seeded on 8-well ibidiTreat polystyrene chamber slides in densities of 5000 and 10,000 cells/cm^2^. The slides were used uncoated as well as coated with 5 µg/cm^2^ Col-I (Merck, Darmstadt, Germany) overnight at 4 °C. 20, 40 and 90 min after seeding, the cells were washed two times with DPBS and fixed with 4% paraformaldehyde in DPBS. Cells seeded to the 8-well slides were stained like for confocal microscopy as mentioned above, only including an additional permeabilization step after fixation with 300 µL 0.1% Triton X100 (Sigma-Aldrich) in DPBS per well. As primary antibody, a rabbit anti-Vinculin (1:250, ab129002, Abcam) and as secondary antibody a donkey anti-rabbit Alexa Fluor 400 conjugate (4 µg/mL, A32790, Thermo Fisher Scientific) were used. The nuclei were counterstained with DAPI and the slides were covered with mounting medium (Ibidi). The experiment was performed twice. Per cell line, treatment and time point between 28 and 39 cells were imaged with an AxioObserver Z1 (Carl Zeiss) and the number of focal adhesions was counted using the FIJI software.

### 4.10. AFM-Adhesion Assay

Cells were seeded 48 h prior to atomic force microscopy (AFM) experiments in culture medium. The medium was replaced after 24 h by culture medium containing 10 mM GlcNAc. As substrate, the lid of a petri dish (Sarstedt, Nümbrecht, Germany) was coated with two 1 cm^2^ drops of 10 µg/mL Col-I (Merck) and 100 µg/mL BSA, respectively, at 4 °C overnight. For AFM force spectroscopy experiments a JPK NanoWizard 1 (JPK Instruments, Berlin, Germany) equipped with a homebuilt temperature unit, ensuring 37 °C during measurements and a CellHesion module (JPK Instruments, Berlin, Germany) with a 100 µm piezo scanner in z-direction was used. The whole setup was mounted on an inverted optical microscope (Axiovert 200, Carl Zeiss, Oberkochen, Germany) placed on an active isolation table (Micro 60, Halcyonics, Göttingen, Germany) inside a homebuilt 1 m^3^ soundproof box to reduce the influence of ambient noise. AFM experiments were carried out using tip-less silicon nitride MLCT cantilevers (Bruker, Karlsruhe, Germany) with a nominal spring constant of ~0.03 N/m. The spring constant was determined for each cantilever individually, using the thermal noise method [43]. Prior to AFM experiments, the cantilevers were coated with 50 µg/mL poly-L-lysine (PLL, Merck, Darmstadt, Germany) at 4 °C overnight. AFM experiments were performed in serum-free HEPES-buffered MEM α medium with 10 mM GlcNAc at 37 °C. Cells were detached from the wells with trypsin for 5 min at 37 °C and transferred to the coated petri dishes and mounted on the AFM to equilibrate at 37 °C for ~10 min. After equilibration, a single cell was gently contacted with the PLL-coated cantilever on the BSA coated area of the petri dish, lifted up and allowed to firmly adhere to the cantilever for one minute. Force-distance curves were recorded within an area of 100 × 100 µm in x-y-direction at 5 × 5 points, on BSA and Col-I coated areas with the same cell. Cells were brought into contact with the respective substrates until the predefined loading force of 100 pN were reached with a vertical cantilever velocity of 5 µm/s. For data analysis a step detection software by Opfer et al. was used [44].

### 4.11. Statistical Analysis

The statistical analysis of all experiments was performed using the GraphPad Prism software (GraphPad Software, San Diego, CA, USA). All graphs and charts show mean values with standard deviation. For the evaluation, a Kruskall-Wallis test with Dunn’s multiple comparison test was used and a *p*-Value < 0.05 was considered as statistically significant.

## Figures and Tables

**Table 1:** PCR Primers.

Primer	Sequence	Annealing Temperature
HAS1_for	5′-GACTCCTGGGTCAGCTTCCTAAG-3′	55 °C
HAS1_rev	5′-AAACTGCTGCAAGAGGTTATTCCT-3′	55 °C
HAS2_for	5′-CATAAAGAAAGCTCGCAACACG-3′	55 °C
HAS2_rev	5′-ACTGCTGAGGAATGAGATCCAG-3′	55 °C
HAS3_for	5′-GACGACAGCCCTGCGTGT-3′	58 °C
HAS3_rev	5′-TTGAGGTCAGGGAAGGAGAT-3′	58 °C
GFP_for	5′-CAAGCTGACCCTGAAGTTCATCTGC-3′	50 °C
GFP_rev	5′-CACGCTGCCGTCCTCGATGTTGTGG-3′	50 °C
GAPDH_for	5′-CAACTACATGGTTTACATGTTC-3′	50 °C
GAPDH_rev	5′-GCCAGTGGACTCCACGAC-3′	50 °C

## Abbreviations

ACTB	actin-beta
AFM	atomic force microscopy
aJ	attojoule
BSA	bovine serum albumin
Col-I	type I collagen
DAPI	4,6-diamidino-2-phenylindole
DPBS	Dulbecco’s phosphate-buffered saline
ECM	extracellular matrix
FGF-2	fibroblast growth factor 2
GAPDH	glyceraldehyde 3-phosphate dehydrogenase
GlcNAc	N-acetyl-D-glucosamine
HA	hyaluronan
HABP	hyaluronic acid binding protein
HAse	hyaluronidase
HAS	hyaluronan synthase
HASes	hyaluronan synthases
hMSCs	human mesenchymal stem cells
kDa	kilodalton
min	minutes
pN	piconewton
RT-PCR	reverse transcriptase-polymerase chain reaction
SCFS	single cell force spectroscopy
Sec	seconds
TCPS	tissue culture polystyrene

## References

[1] Laurent T.C. (1987). Biochemistry of hyaluronan. Acta Otolaryngol. Suppl..

[2] Toole B.P. (2004). Hyaluronan: From extracellular glue to pericellular cue. Nat. Rev. Cancer.

[3] Lee G.M., Johnstone B., Jacobson K., Caterson B. (1993). The dynamic structure of the pericellular matrix on living cells. J. Cell Biol..

[4] Qu C., Rilla K., Tammi R., Tammi M., Kroger H., Lammi M.J. (2014). Extensive CD44-dependent hyaluronan coats on human bone marrow-derived mesenchymal stem cells produced by hyaluronan synthases HAS1, HAS2 and HAS3. Int. J. Biochem. Cell Biol..

[5] Cohen M., Kam Z., Addadi L., Geiger B. (2006). Dynamic study of the transition from hyaluronan- to integrin-mediated adhesion in chondrocytes. EMBO J..

[6] Itano N., Sawai T., Yoshida M., Lenas P., Yamada Y., Imagawa M., Shinomura T., Hamaguchi M., Yoshida Y., Ohnuki Y. (1999). Three isoforms of mammalian hyaluronan synthases have distinct enzymatic properties. J. Biol. Chem..

[7] Itano N., Kimata K. (2002). Mammalian hyaluronan synthases. IUBMB Life.

[8] Weigel P.H., DeAngelis P.L. (2007). Hyaluronan synthases: A decade-plus of novel glycosyltransferases. J. Biol. Chem..

[9] Knudson C.B., Knudson W. (1993). Hyaluronan-binding proteins in development, tissue homeostasis, and disease. FASEB J..

[10] Itano N. (2008). Simple primary structure, complex turnover regulation and multiple roles of hyaluronan. J. Biochem..

[11] Geiger B., Bershadsky A., Pankov R., Yamada K.M. (2001). Transmembrane crosstalk between the extracellular matrix--cytoskeleton crosstalk. Nat. Rev. Mol. Cell Biol..

[12] Cohen M., Joester D., Geiger B., Addadi L. (2004). Spatial and temporal sequence of events in cell adhesion: From molecular recognition to focal adhesion assembly. Chembiochem.

[13] Zaidel-Bar R., Cohen M., Addadi L., Geiger B. (2004). Hierarchical assembly of cell-matrix adhesion complexes. Biochem. Soc. Trans..

[14] Zimmerman E., Geiger B., Addadi L. (2002). Initial stages of cell-matrix adhesion can be mediated and modulated by cell-surface hyaluronan. Biophys. J..

[15] Petit V., Thiery J.P. (2000). Focal adhesions: Structure and dynamics. Biol. Cell.

[16] Zamir E., Geiger B. (2001). Molecular complexity and dynamics of cell-matrix adhesions. J. Cell Sci..

[17] Hynes R.O. (2002). Integrins: Bidirectional, allosteric signaling machines. Cell.

[18] Cohen M., Klein E., Geiger B., Addadi L. (2003). Organization and adhesive properties of the hyaluronan pericellular coat of chondrocytes and epithelial cells. Biophys. J..

[19] Hanein D., Sabanay H., Addadi L., Geiger B. (1993). Selective interactions of cells with crystal surfaces. Implications for the mechanism of cell adhesion. J. Cell Sci..

[20] Hanein D., Geiger B., Addadi L. (1994). Differential adhesion of cells to enantiomorphous crystal surfaces. Science.

[21] Friedenstein A.J., Chailakhjan R.K., Lalykina K.S. (1970). The development of fibroblast colonies in monolayer cultures of guinea-pig bone marrow and spleen cells. Cell Tissue Kinet..

[22] Caplan A.I. (1991). Mesenchymal stem cells. J. Orthop. Res..

[23] Prockop D.J. (1997). Marrow stromal cells as stem cells for nonhematopoietic tissues. Science.

[24] Pittenger M.F., Discher D.E., Peault B.M., Phinney D.G., Hare J.M., Caplan A.I. (2019). Mesenchymal stem cell perspective: Cell biology to clinical progress. NPJ Regen. Med..

[25] Solis M.A., Chen Y.H., Wong T.Y., Bittencourt V.Z., Lin Y.C., Huang L.L. (2012). Hyaluronan regulates cell behavior: A potential niche matrix for stem cells. Biochem. Res. Int..

[26] Kota D.J., Prabhakara K.S., Cox C.S., Olson S.D. (2014). MSCs and hyaluronan: Sticking together for new therapeutic potential?. Int. J. Biochem. Cell Biol..

[27] Bocker W., Yin Z., Drosse I., Haasters F., Rossmann O., Wierer M., Popov C., Locher M., Mutschler W., Docheva D. (2008). Introducing a single-cell-derived human mesenchymal stem cell line expressing hTERT after lentiviral gene transfer. J. Cell Mol. Med..

[28] Rilla K., Siiskonen H., Spicer A.P., Hyttinen J.M., Tammi M.I., Tammi R.H. (2005). Plasma membrane residence of hyaluronan synthase is coupled to its enzymatic activity. J. Biol. Chem..

[29] Sariisik E., Docheva D., Padula D., Popov C., Opfer J., Schieker M., Clausen-Schaumann H., Benoit M. (2013). Probing the interaction forces of prostate cancer cells with collagen I and bone marrow derived stem cells on the single cell level. PLoS ONE.

[30] Sariisik E., Popov C., Muller J.P., Docheva D., Clausen-Schaumann H., Benoit M. (2015). Decoding Cytoskeleton-Anchored and Non-Anchored Receptors from Single-Cell Adhesion Force Data. Biophys. J..

[31] Benoit M., Gabriel D., Gerisch G., Gaub H.E. (2000). Discrete interactions in cell adhesion measured by single-molecule force spectroscopy. Nat. Cell Biol..

[32] Polzer H., Volkmer E., Saller M.M., Prall W.C., Haasters F., Drosse I., Wilhelmi A., Mutschler W., Schieker M. (2014). Comparison of different strategies for in vivo seeding of prevascularized scaffolds. Tissue Eng. Part C Methods.

[33] Kavanagh D.P., Robinson J., Kalia N. (2014). Mesenchymal stem cell priming: Fine-tuning adhesion and function. Stem. Cell Rev. Rep..

[34] Jung E.M., Kwon O., Kwon K.S., Cho Y.S., Rhee S.K., Min J.K., Oh D.B. (2011). Evidences for correlation between the reduced VCAM-1 expression and hyaluronan synthesis during cellular senescence of human mesenchymal stem cells. Biochem. Biophys. Res. Commun..

[35] Shimabukuro Y., Ueda M., Ichikawa T., Terashi Y., Yamada S., Kusumoto Y., Takedachi M., Terakura M., Kohya A., Hashikawa T. (2005). Fibroblast growth factor-2 stimulates hyaluronan production by human dental pulp cells. J. Endod..

[36] Calabro A., Oken M.M., Hascall V.C., Masellis A.M. (2002). Characterization of hyaluronan synthase expression and hyaluronan synthesis in bone marrow mesenchymal progenitor cells: Predominant expression of HAS1 mRNA and up-regulated hyaluronan synthesis in bone marrow cells derived from multiple myeloma patients. Blood.

[37] Brinck J., Heldin P. (1999). Expression of recombinant hyaluronan synthase (HAS) isoforms in CHO cells reduces cell migration and cell surface CD44. Exp. Cell Res..

[38] Kultti A., Rilla K., Tiihonen R., Spicer A.P., Tammi R.H., Tammi M.I. (2006). Hyaluronan synthesis induces microvillus-like cell surface protrusions. J. Biol. Chem..

[39] Ponta H., Sherman L., Herrlich P.A. (2003). CD44: From adhesion molecules to signalling regulators. Nat. Rev. Mol. Cell Biol..

[40] Nilsson S.K., Haylock D.N., Johnston H.M., Occhiodoro T., Brown T.J., Simmons P.J. (2003). Hyaluronan is synthesized by primitive hemopoietic cells, participates in their lodgment at the endosteum following transplantation, and is involved in the regulation of their proliferation and differentiation in vitro. Blood.

[41] Ellis S.L., Grassinger J., Jones A., Borg J., Camenisch T., Haylock D., Bertoncello I., Nilsson S.K. (2011). The relationship between bone, hemopoietic stem cells, and vasculature. Blood.

[42] Schindelin J., Arganda-Carreras I., Frise E., Kaynig V., Longair M., Pietzsch T., Preibisch S., Rueden C., Saalfeld S., Schmid B. (2012). Fiji: An open-source platform for biological-image analysis. Nat. Methods.

[43] Butt H.-J., Jaschke M. (1995). Calculation of thermal noise in atomic force microscopy. Nanotechnology.

[44] Opfer J., Gottschalk K.E. (2012). Identifying discrete states of a biological system using a novel step detection algorithm. PLoS ONE.

